# *QuickStats:* Percentage* of Currently Employed Adults Who Have Paid Sick Leave,^†^ by Industry^§^ — National Health Interview Survey, 2009 and 2018^^¶^^

**DOI:** 10.15585/mmwr.mm6834a6

**Published:** 2019-08-30

**Figure:**
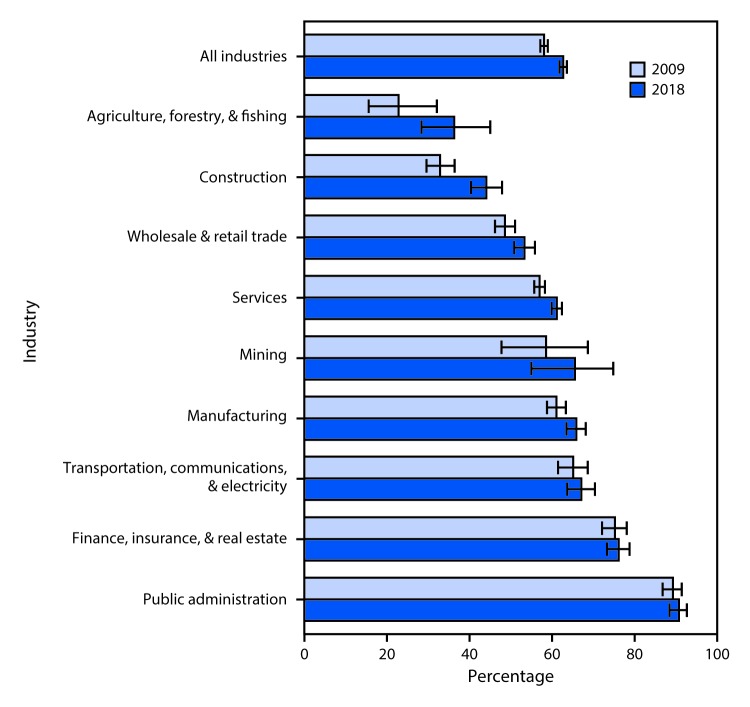
The percentage of all currently employed workers with access to paid sick leave increased from 57.8% in 2009 to 62.4% in 2018. By industry, the percentage increased for workers in construction (32.7% to 43.9%), wholesale & retail trade (48.3% to 53.1%), services (56.7% to 60.8%), and manufacturing (60.7% to 65.5%). In 2018, fewer than half of workers in agriculture, forestry, and fishing and construction industries had access to paid sick leave compared to approximately 90% of workers in public administration.

**Source:** National Health Interview Survey, 2009–2018. https://www.cdc.gov/nchs/nhis.htm.

